# Nitrifying trickling filters and denitrifying bioreactors for nitrogen management of high-strength anaerobic digestion effluent

**DOI:** 10.1016/j.chemosphere.2018.03.137

**Published:** 2018-08

**Authors:** Aaron A. Forbis-Stokes, Lucas Rocha-Melogno, Marc A. Deshusses

**Affiliations:** a Department of Civil & Environmental Engineering, Duke University, Durham, NC, USA; b Duke Global Health Institute, Duke University, Durham, NC, USA

**Keywords:** Nitrification, Denitrification, Trickling filter, Submerged anaerobic filter, Sanitation, Onsite treatment

## Abstract

The treatment of high-strength anaerobic digester effluent in laboratory-scale trickling filters for nitrification and then anaerobic filters for denitrification is reported. Five media types were investigated in the trickling filters: biochar, granular activated carbon (GAC), zeolite, Pall rings, and gravel. Three media were tested in five denitrifying filters: sand (S), bamboo wood chips (B), eucalyptus wood chips (E), bamboo with sand (B+S), and eucalyptus with sand (E+S). The different wood chips served as a supplemental electron donor for denitrification. From six months of operation, biochar, GAC, zeolite, Pall rings, and gravel media had turbidity (NTU) removal efficiencies of 90, 91, 77, 74, and 74%, respectively, and ammonia removal efficiencies of 83, 87, 85, 30, and 80%, respectively, which was primarily by nitrification to nitrate. For the anaerobic filters, S, B, B+S, E, and E+S had nitrate removal efficiencies of 30, 66, 53, 35, and 35%, and turbidity removal efficiencies of 88, 89, 84, 89, and 88%, respectively. Biochar and bamboo were selected as the best combination of media for trickling filter and anaerobic filter sequential treatment. Based on an average initial influent of 600 mg NH_3_-N L^−1^, 50 mg NO_3_-N L^−1^, and 980 NTU, the biochar filter's effluent would be 97 mg NH_3_-N L^−1^, 475 mg NO_3_-N L^−1^, and 120 NTU. The bamboo filter's final effluent would be 82 mg NH_3_-N L^−1^, 157 mg NO_3_-N L^−1^, and 13 NTU, which corresponds to 63% removal of total N and 99% removal of turbidity. These filter media thus present a simple option for sustainable post-treatment for nitrogen management and effluent polishing in low-resources settings.

## Highlights

• High-strength anaerobic digester effluent was treated in biological filters.
• Trickling filters packed with biochar, GAC, and zeolite showed high removal of NH_3_-N.
• Trickling filter effluent was denitrified in submerged filters packed with different media.
• Bamboo can be an effective electron donor for denitrification while acting as filter media.
• High removal of N and turbidity was achieved using the proposed sequential treatment.

## Graphical abstract

**Figure:**
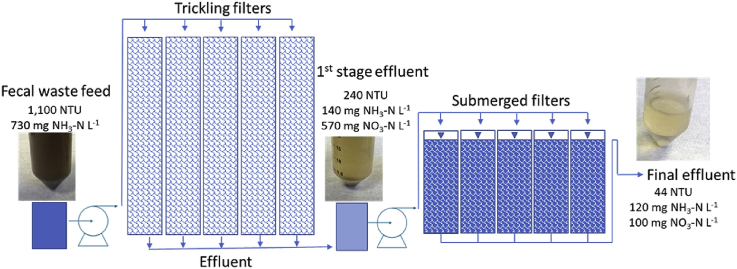

## 1 Introduction

The need for onsite sanitation and fecal sludge management is considerable as 2.7 billion people worldwide currently rely on these technologies, and the number is expected to grow to 5 billion by 2030 (Strande, 2014). Onsite sanitation is desirable as the centralized sewer-based collection and treatment systems existing in developed nations are too costly, too complex, and use too much energy to implement in poor and less developed countries (Lalander et al., 2013; Mara, 2013). While influent concentrations at conventional sewage treatment plants are generally in the range of 300–2000 mg_COD_ L^−1^, <1% TSS, and 50–250 mg_TN_ L^−1^, fecal sludge reaching onsite treatment systems can have as much as 30,000–100,000 mg_COD_ L^−1^, 3–7% TSS, and 3000–7000 mg_TN_ L^−1^ (where COD is for chemical oxygen demand and TN is total nitrogen) (Strande et al., 2014). Further, most existing onsite sanitation solutions (e.g. anaerobic digestion, stabilization ponds, drying beds, etc.) only consider organic removal and pathogen destruction but do not address nutrients. In conventional sewage treatment, nutrient removal is a cumbersome, expensive step. Therefore, there is a need for alternative methods for efficient onsite nutrient recovery or removal which require minimal, if any, external resources.

Application of trickling filters (TF) for the post-treatment of anaerobic reactors handling sewage is a relatively recent interest (Chernicharo and Nascimento, 2001; Tandukar et al., 2006; von Sperling and Chernicharo, 2005). Trickling filters can achieve biochemical oxygen demand (BOD) and solids removal as well as nitrification, the biological oxidation of ammonia to nitrite and nitrate (Daigger and Boltz, 2011). Trickling filters are therefore worth consideration for onsite treatment.

Studies on trickling filter for post-anaerobic digestion treatment are limited (Khan et al., 2011). Chernicharo and Nascimento (2001) operated a trickling filter filled with 4–6 cm furnace slag at organic loading rates of 0.3–3.9 kg_BOD_ m^−3^ d^−1^. This filter treated effluent from a UASB and reached BOD removals in the range of 50–60% but authors did not monitor NH_3_ removal. Using pebbles with an effective size of 5.6 mm for the treatment of anaerobically digested dairy manure, Xia et al. (2012) achieved average removal of 71% of soluble BOD (sBOD) and 75% NH_3_-N for influent containing 692 mg_sBOD_ L^−1^ and 1210 mg NH_3_-N L^−1^. Vieira et al. (2013) used a novel TF design to achieve 32% BOD and 31% NH_3_-N removal from UASB effluent starting with 72 mg_BOD_ L^−1^ and 29 mg NH_3_-N L^−1^ under OLR and HLR of 0.08 kg_BOD_ m^−3^ d^−1^ and 1.1 m^3^ m^−2^ d^−1^, respectively. In a downflow hanging sponge (DHS) reactor, a setup similar to TF, Tandukar et al. (2006) found 83–87% BOD removal and 70–73% NH_3_ removal (inlet concentration up to 40 mg NH_4_-N L^−1^) while operating at 0.67 to 1.24 kg_BOD_ m^−3^ d^−1^, respectively. Most recent work with this reactor type achieved removals of 87% and 83% of BOD and NH_3_-N, respectively at a loading of 1.11 kg_BOD_ m^−3^ d^−1^ with influent concentrations of 93 mg_BOD_ L^−1^ and 24.6 mg NH_4_-N L^−1^ (Onodera et al., 2014). It should be noted that most of these previous studies dealt with domestic sewage where influents contained less than 50 mg NH_3_-N L^−1^ whereas treating anaerobic digester effluent from onsite fecal sludge treatment will require handling NH_3_-N concentrations on the order of 1–5 g L^−1^.

Ideal media characteristics for TF are high specific surface area for biofilm attachment, high porosity to prevent clogging and allow for easy air flow, low cost, and long durability (Tchobanoglous et al., 2003; Daigger and Boltz, 2011). Rocks have been traditionally used though today, plastic media have become more common allowing high organic loadings, and effective biofilm wetting and aeration. Zeolite is a medium of interest in trickling filters due to its adsorption properties. Aiyuk et al. (2004) used a zeolite column following a UASB reactor treating domestic wastewater and achieved 99% removal of NH_4_^+^. The proposed ammonium removal mechanism was ion exchange as interstitial spaces in the zeolite framework allow replacement of Na^+^, K^+^, and Ba^2+^ cations with NH_4_^+^. One interesting finding was the ability to recharge the zeolite's ion exchange capabilities through nitrification and that the recharged zeolite displayed similar performance as fresh zeolite (Aiyuk et al., 2004). The process of adsorption, biological degradation, and nitrification was later explored by various investigators (Luo et al., 2014; Masunga et al., 2007; Yidong et al., 2012). In particular, Luo et al. (2014) used zeolite in a trickling filter with influent concentrations of 265 mg_COD_ L^−1^ and 41.1 mg NH_3_ L^−1^ and achieved 94.7% and 90.4% removal, respectively.

The use of granular activated carbon (GAC) as a biofilm carrier was introduced in drinking water treatment and showed improvements over adsorption-only processes in that media lifespan was extended such that it did not require regeneration (Servais et al., 1994). In greywater trickling filter applications, Dalahmeh et al. (2012) achieved 97% BOD and 98% total nitrogen reduction of an artificial greywater containing 425 mg_BOD5_ L^−1^ and 75 mg total N (TN) L^−1^. There are no known studies of the use of GAC as a trickling filter media for domestic wastewater.

Biochar is a medium of interest due to its physical characteristics, sustainable production, and global availability. Biochar presents a low cost alternative to GAC. Its physical properties are highly variable depending on substrate and pyrolysis temperature with ranges for surface area and bulk density of 50–500 m^2^ g^−1^ and 1.5–2.0 g cm^−3^, respectively (Downie et al., 2012). In the treatment of artificial greywater with 1390 mg_COD_ L and 95 mg TN L^−1^, Berger (2012) found 99.2% and 99.1% COD and 96.6% and 90.9% TN removal efficiencies with GAC and biochar, respectively. Li et al. observed similar performance with biochar and achieved up to 91.4% removal of NH_4_^+^-N under loading concentrations of 100–150 mg NH_4_^+^-N L^−1^ while operating at a 72 h retention time (Li et al., 2016). Like GAC, there are no known studies of the use of biochar as a trickling filter media for domestic wastewater.

The above review suggests that treatment of digester effluent treatment in trickling filters shows great promise for reducing organic and solids content while oxidizing ammonia into nitrate. This process should improve the effluent quality to make it more likely to be reused as a fertilizer (cleaner, and nitrate being more desirable than ammonia). Additionally, the trickling filter effluent could be denitrified if total nitrogen removal is necessary, making it possible to consider various water reuse scenarios. Thus, potential low-cost means for denitrification of nitrified digester effluent are discussed next.

The nitrate and nitrite produced from nitrification of anaerobically digested fecal sludge can be removed by denitrification, the anaerobic biological reduction of nitrate and nitrite to N_2_ gas. Submerged fixed beds have been successfully used for denitrification. The denitrification process requires an electron donor which can be residual BOD or provided extraneously. Generally the electron donor is the rate-limiting substrate, and a requirement of 4 g BOD_L_/g NO_3_^−^-N is normally assumed for complete denitrification to nitrogen gas (Rittmann and McCarty, 2001). Effective trickling filter operation is expected to remove COD; however, particulate and slowly biodegradable COD that passes through the trickling filter may be utilized for denitrification (Nakhla and Farooq, 2003). An exogenous electron donor may be necessary if not enough BOD remains in the stream undergoing treatment. Methanol and acetate are commonly supplied as electron donors in conventional wastewater treatment plants, but this practice would probably be cost- and resource-prohibitive for onsite fecal sludge treatment.

An alternative method of providing an electron donor is by using a carbon-based filter medium. The use of wood chips as a denitrifying filter media has gained attention recently, typically for stormwater and agricultural drainage (Addy et al., 2016; Bock et al., 2015, 2016; Cameron and Schipper, 2010; Christianson et al., 2017; Lynn et al., 2015a, 2015b; Pluer et al., 2016; Schipper et al., 2010c; Schipper et al., 2010a). These studies have relied on woodchips to support denitrification of waste streams with up to 30 mg NO_3_-N L^−1^ and at rates of 2–22 g N m^−3^ d^−1^ (Schipper et al., 2010b). The highest nitrate loading concentration and removal rate of these studies was found by Lepine et al. (2016) at 80 mg NO_3_-N L^−1^ and 39 g N m^−3^ d^−1^, respectively. Two studies evaluated the use of wood chips in denitrifying onsite sewage effluent (Rambags et al., 2016; Robertson et al., 2005) with similar influent nitrate loading (∼30 mg NO_3_-N L^−1^) and removal (87–99%). In an evaluation of alternative nitrogen management technologies for onsite sewage treatment, a simple single pass sand filter followed by a wood chip biofilter outperformed more complicated technologies (Oakley et al., 2010). Still, few studies have evaluated wood chips for nitrogen treatment of onsite sewage. These studies have experimented with different types of wood chips from hardwoods or softwoods such as eucalyptus, pine, willow, or mixed, but none so far has used bamboo. Bamboo is a wood source that is readily available in tropical regions of the world and can be sustainably grown. Study on three types of bamboo found that 51–52% of dry weight was carbon and 14–17% was fixed carbon (Oak Ridge National Laboratory, 2008).

In this paper, a sequential trickling filter and submerged attached growth filter for nitrification-denitrification of anaerobic digester effluent is presented. The objective of this study was to evaluate five media types for simultaneous removal of COD, turbidity, and nitrification of ammonia from high-strength anaerobic digester effluent. In the second step three media types were evaluated for denitrification without supplementing an external donor, relying on either the remaining COD in the trickling filter effluent (sand filter) and/or the COD provided by the wood media themselves (bamboo and eucalyptus filters). This is the first study to assess these media types for sequential nitrification-denitrification of high-strength digester effluent.

## 2 Materials & methods

### 2.1 Filter materials

Five different media types were selected for the trickling filter: biochar (thereafter abbreviated BC), granular activated carbon (GAC), zeolite (abbreviated Zeo), Pall rings (PR), and gravel (Gr). The submerged attached growth filters for denitrification included sand (S), chipped bamboo (B), and chipped eucalyptus (E). Biochar was chosen due to its sustainable production and availability. Because previous applications of biochar as a media for biofiltration of wastewater were not found, GAC was selected for comparison to a similar previously studied medium. Zeolite was chosen to include a medium with ammonium adsorption capabilities in a biological filter application. Pall rings and gravel were chosen for comparison to engineered (Pall rings) and historical (gravel) alternatives.

For the denitrifying filters, sand was selected to determine if denitrification could take place without external supply of electron donors, eucalyptus was used for comparison to previous studies, and bamboo was used as a potential alternative, more sustainable, source of wood chips. Additionally, sand was mixed with wood chips (both bamboo and eucalyptus) in separate columns to see if the combination would yield improved turbidity and nitrate removal.

The biochar was derived from pine pellets and manufactured at 900 °C for 1 h. GAC was sized at 4 × 8 mesh and derived from coconut shell. Jaeger Pall rings were in polypropylene and measured 16 mm (5/8”) in diameter. Biochar was provided by the group of Prof. Linden at the University of Colorado-Boulder. GAC, zeolite, and Pall rings were purchased from online vendors. Quarried, washed, and dried sand was sourced from Sakrete, and gravel was sourced from the Martin Marietta Plant in Durham, NC. Bamboo (*Phyllostachys edulis*) was sourced locally in North Carolina and passed through a wood shredder. Eucalyptus chips were purchased from a vendor in Florida. In both cases, only wood chips passing through a sieve with 9.5 mm openings were used in the filter.

Five sequential nitrification and denitrification filters were constructed. All filters were constructed using 10 cm inner diameter clear PVC pipe. Columns were wrapped with aluminum foil to prevent the growth of photosynthetic organisms. The column:media diameter ratio was at least 20:1 for all columns (except for Pall rings and wood chips) in order to minimize wall effects in the filters.

Trickling filter columns were filled with media to a depth of 100 cm (total volume of 8.0 L, each) and were manually shaken every 20 cm to ensure uniform packing. Media were held using a plastic mesh at the bottom of the column. Natural aeration was induced through air inlet ports (one 13 mm for sampling, three 3 mm for air) drilled into the fittings below the filter bed as well as keeping the top of the bed open to the atmosphere.

Submerged filter columns were filled with sand, bamboo, eucalyptus, bamboo with sand (B+S), and eucalyptus with sand (E+S), respectively, to a depth of 50 cm (total volume of 4.0 L). Mixed wood chip and sand columns were filled in 10 cm increments until reaching the full height of 50 cm. Wood chips were added first, and then sand was added while shaking the column to minimize heterogeneous void space.

Media physical characteristics were determined through a sieve analysis on a mechanical shaker at 30 rpm for 10 min. Results are shown in Table 1 and in Supplementary Information Table A.1. Each medium was passed through 6.3, 4.75, 3.35, 2.36, and 1.18 mm meshes to determine effective grain size (*D*_*10*_) and uniformity coefficient (*D*_*60*_*/D*_*10*_). Bulk density, particle density, and porosity were determined for each column. Bulk density (*ρ*_*B*_) was determined as the mass of media filling the column volume, and particle density (*ρ*_*p*_) was determined using the volume of deionized water displaced by 25 g of each sample. Porosity (*p*) was calculated with equation (1).

(1) $$p=100\times \left(1-\frac{\rho_{B}}{\rho_{p}}\right)$$

**Table 1:** Effective size, uniformity coefficient, and bulk density of the media used in the trickling filters and in the anaerobic filters.

	Effective size (mm)	Uniformity Coefficient (−)	Bulk Density (kg/m^3^)	Particle Density (kg/m^3^)	Bed Porosity (%)
Biochar	4.9	1.20	355	1263	72
GAC	2.5	1.44	486	1667	71
Zeolite	2.8	1.68	804	1668	52
Pall Rings	15.9	1.00	110	927	88
Gravel	5.6	1.39	1376	2781	51
Sand (S)	2.5	1.44	1405	2938	52
Bamboo (B)	2.0	3.40	244	907	73
B+S	2.3	1.96	788	2308^a^	66
Eucalyptus (E)	1.6	3.44	267	752	65
E+S	2.1	2.06	644	2198^a^	71

a Weighed average for the mixture.

### 2.2 Fecal waste treated

Minimally diluted anaerobically digested human fecal sludge was not available in sufficient quantities for these experiments. Thus, a suitable simulant was sought. Expected BOD and total nitrogen concentrations are 1–5 g_BOD_ L^−1^ and 1–3 g N L^−1^. The effluent from an intermittently aerated basin treating anaerobically digested swine manure system was selected as a mimic for human fecal digestate (Xu et al., 2016). Previous monitoring of this system had shown effluent characteristics to be in the target range for COD and TN; however, further analysis revealed that most of the COD was recalcitrant (COD:BOD = ∼5:1) which may have resulted in low heterotrophic activity in the trickling filters and low availability of electron donor for denitrification in the anaerobic filters. The effluent basin was known to contain both nitrifying and denitrifying bacteria and was used to inoculate the trickling and submerged filters. During the experiments, the swine waste was added directly to the trickling filters. The effluent from all trickling filters was pooled together and collected in a 5 L flask that was continuously mixed, and this liquid was used as the influent for submerged filters.

Each trickling and anaerobic filter was fed 1.35 L d^−1^ using peristaltic pumps connected to timers. Feeding was applied at 27 mL min^−1^ for 5 min at the beginning of each chosen hour from 6:00–8:05, 11:00–13:05, and 17:00–20:05. This feeding pattern was an approximation of the expected daily flow pattern in an on-site fecal sludge treatment system. The daily average hydraulic loading rate (HLR) on each filter was $\text{0}\text{.}17\;{\text{m}}^{3}\;{\text{m}}_{\text{cross}-\text{section}}^{-2}\;{\text{d}}^{-1}$, the organic loading rate (OLR) was 0.38 and $\text{0}\text{.}23\;\text{kg}\;\text{COD}\;{\text{m}}_{\text{reactor}}^{-3}\;{\text{d}}^{-1}$ for the trickling filters and the anaerobic filters, respectively and nitrogen loading rate was $\text{0}\text{.}3-1\text{.0}\;\text{g}\;{\text{m}}_{\text{media}}^{-3}\;{\text{d}}^{-1}$. Empty bed residence time (EBRT) was 6 days for the trickling filters and 3 days for the submerged filters. However, the trickling filters have a liquid hold-up of about 5–10% and thus the true residence time in the TFs is more in the range of 7–15 h. These design characteristics were chosen so that a conservative estimate for performance (assuming a media surface area of 500 m^2^ m^-3^) would remain under previously established maximum operational parameters for simultaneous nitrification and BOD removal: 80 m^3^ m^−2^ d^−1^, 0.5 kg_BOD_ m^−3^ d^−1^, and $1\;\text{g}\;\text{N}\;{\text{m}}_{\text{media}}^{\mathrm{-2}}\;{\text{d}}^{-1}$ (Tchobanoglous et al., 2003). Due to the low flow rate, a uniform distribution of the substrate at the top of the filter was not achieved. However, uniform liquid distribution was achieved within the depth of the media and was confirmed in hydraulic tests conducted with water. Filters were wetted with tap water for two weeks before substrate addition. Filters were operated at ambient laboratory temperatures (20 ± 2 °C).

Swine farm effluent used as substrate was undiluted for the first 8 weeks of operation (Period I). The high concentration of suspended solids (approximately 2000 mg L^−1^) contributed to operational issues (plugging and issues with pumping thick slurries), thus the substrate was diluted 1:1 with tap water for the remainder of the study (Periods II and III). Average COD and TN concentrations during these periods were 3040 and 1830 mg_COD_ L^−1^ and 1790 and 790 mg N L^−1^, respectively. The denitrifying filter study began after the trickling filter study (day 225, Period II) and ran for 120 days. Operating conditions did not change for the denitrifying filters, so this study is not divided into periods as in the trickling filter study.

### 2.3 Analytical and experimental protocols

The true hydraulic retention time was determined in both reactor types from tracer studies using conductivity probes (Vernier, Beaverton, OR). A step change from water to trickling filter substrate was used due to high conductivity of substrate (∼20,000 μs/cm). Conductivity probes were placed in the low volume vessels (∼50 mL) that collected the TF effluent before it overflowed into the secondary container. Substrate for submerged filters had a lower conductivity, and thus a NaCl solution (20,000 μs/cm) was pumped into these filters for one day. Conductivity was measured in the low volume vessels (∼50 mL) that collected filter effluent before it left the system. The retention time in the trickling filters was determined as the time at which 50% of the peak conductivity was measured. In the submerged filters, retention time was determined as the time in which 50% of the tracer input was recovered in the effluent.

Samples were taken weekly for the study duration from the outlet of each filter and at the mid-depth of the submerged filter. Total NH_3_-N, NO_2_^−^-N, NO_3_^−^-N, and COD were measured using Hach kits. Dissolved oxygen (DO) and pH were measured using digital meters (Hach HQD portable meter with Intellical LDO101 DO sensor, Loveland, CO; and Oakton Instruments Ion 510 series pH meter, Vernon Hills, IL; respectively), and turbidity was measured using EPA Method 180.1. Periodic tests of BOD_5_ and total suspended solids were performed according to EPA Methods 405.1 and 160.2, respectively.

Filters were not backwashed during this study. After 260 days (end of Period II), plugging at the top of the trickling filters had become a regular issue. Feeding was stopped at this time, and media was removed from each filter and washed with tap water. Period III began at this time (day 274). In Period III fresh biochar (BC-new) and fresh GAC (GAC-new) replaced Zeo and PR filters while washed BC, GAC, and G media were reused. Operating conditions in Period III were the same as in Period II.

COD release from wood chips was evaluated in both a batch and in a column experiment. For the batch experiment, 10 g of either bamboo or eucalyptus wood chips were added into 250 mL flasks filled with 100 mL DI water, 100 mL trickling filter effluent, and 1 mL of sludge for inoculation. Separate flasks were filled with the same solution without wood chips as a control. All flasks were prepared in triplicate and incubated on a shaker table at room temperature (22 °C) without exposure to light. Liquid samples were taken with a syringe (2 mL) to measure COD and NO_3_-N concentrations in the solution. A manometer was used to measure the change of pressure in the headspace. Samples were taken at 12 h, 1, 2, 3, and 6 days and then weekly after the start of the experiment. A COD mass balance was performed to determine how the COD released from wood chips was dissolved into solution, consumed by denitrifiers, or used for CH_4_ production as measured in the headspace. The total COD release was calculated using the following equations:

(2) $$COD_{solution}\left[g\right]=V_{solution}\left[L\right]\;\text{*}\;COD_{measured}\left[\frac{g}{L}\right]$$

(3) $$COD_{NO3}\left[g\right]=V_{solution}\left[L\right]\;\text{*}\;\left(NO_{3,initial}-NO_{3,final}\right)\left[\frac{g}{L}\right]\;\text{*}\;4\left[\frac{g\;COD}{g\;NO_{3}}\right]$$

(4) $$COD_{CH4}\left[g\right]=\text{\%}CH_{4}\;\text{*}\;\frac{P\left[Pa\right]\text{*}V_{headspace}\left[m^{3}\right]}{R\left[\frac{m^{3}\text{*}Pa}{K\text{*}mol}\right]\text{*}T\left[K\right]}\;\text{*}\;64\left[\frac{g\;COD}{\text{mol}\;\text{CH}4}\right]$$

(5) $$COD_{total}\left[g\right]=COD_{solution}\left[g\right]+COD_{NO3}\left[g\right]+COD_{CH4}\left[g\right]$$

where V_liquid_ is the volume of solution on the day of sampling, P is pressure of gas in headspace, %CH_4_ the fraction of methane in the headspace, V_headspace_ is the volume of gas headspace on the day of sampling, R is the ideal gas law constant 8.314 [m^3^*Pa/K*mol], and T is temperature in Kelvin (295 K). The conversion of g NO_3_-N to g COD is based on the relationship of g COD expected to denitrify NO_3_-N to N_2_ (Rittmann and McCarty, 2001). The %CH_4_ was determined by GC-TCD analysis (SRI 8610C, California). Only gauge pressure was considered to calculate number of moles of CH_4_ produced, assuming atmospheric pressure remained constant during the experiment. The conversion of CH_4_ to COD is based on the empirical COD of CH_4_, 4 mol O_2_ to oxidize 1 mol CH_4_ and 16 g/mol CH_4_ resulting in 64 g COD/mol CH_4_.

A similar experiment was performed to determine COD released by operating columns with fresh bamboo and eucalyptus. Filters were fed trickling filter effluent (300 mg COD L^−1^ and 400 mg NO_3_-N L^−1^) for the first three days. The feed was then switched to a solution of DI water and KNO_3_ (0 mg COD L^−1^ and 400 mg NO_3_-N L^−1^) so that all denitrification would be due to COD released from wood chips. COD and NO_3_-N were determined in the inlet, mid-depth, and outlet of the columns at the start and then 1, 2, 3, 6, 9, and 12 days after the start and then weekly until the outlet concentration of nitrate equaled the inlet concentration. The COD released by wood chips was determined by a mass balance, assuming steady-state conditions after the first three days (no COD accumulation) where C_in_ and C_out_ were COD concentrations measured in inlet and outlet, respectively, Q was substrate flow rate (1.35 L d^−1^), and t was time step between sampling days [d]. The following mass balance was solved for wood chip release, WC_release_, in g COD.

(6) $$0\;\left[g\;COD\right]=\left(C_{in}-C_{out}\right)\;\text{*}\;Q\;\text{*}\;t+WC_{release}-\left(NO_{3,in}-NO_{3,out}\right)\;\text{*}\;Q\;\text{*}\;t\;\text{*}\;4\left[\frac{g\;COD}{g\;NO_{3}}\right]$$

(7) $$WC_{release}\left[g\;COD\right]=-\left(C_{in}-C_{out}\right)\;\text{*}\;Q\;\text{*}\;t+\left(NO_{3,in}-NO_{3,out}\right)\;\text{*}\;Q\;\text{*}\;t\;\text{*}\;4\left[\frac{g\;COD}{g\;NO_{3}}\right]$$

## 3 Results

### 3.1 Nitrifying filters

Tracer study results found that the actual retention time in BC, GAC, Zeo, PR, and Gr filters was 66.1, 88.1, 61.0, 50.5, and 65.2 h, respectively. The ratio of the actual retention time to EBRT (6 days) for these filters were 0.46, 0.61, 0.42, 0.35, and 0.45, respectively. Table 2 shows average values for COD, pH, and turbidity for filter substrate (influent) and concentrations for each filter's effluent during Periods II & III.

**Table 2:** Average influent and effluent COD, pH, and turbidity, and changes (Δ) from inlet to outlet during Period II (days 56–260) and Period III (days 274 onwards).

	COD (mg/L)			pH (−)			Turbidity (NTU)		
	Avg.	St. Dev.	*Δ (%)*	Avg.	St. Dev.	*Δ (*pH *units)*	Avg.	St. Dev.	*Δ (%)*
Influent – II	1863	904		8.2	0.2		1146	770	
Influent – III	1919	803		7.5	0.6		814	357	
BC – II	818	367	−*56%*	5.4	1.2	−*2.8*	239	154	−*87%*
BC – III	398	229	−*79%*	6.1	1.0	−*0.6*	53	75	−*94%*
BC new - III	747	468	−*61%*	5.5	1.5	−*2.0*	141	185	−*83%*
GAC – II	716	486	−*62%*	6.0	1.2	−*2.2*	217	214	−*88%*
GAC – III	441	379	−*77%*	6.8	1.0	−*0.7*	135	134	−*83%*
GAC new – III	483	397	−*75%*	6.2	1.1	−*1.3*	129	157	−*84%*
Zeo – II	1445	578	−*22%*	6.7	0.7	−*1.5*	493	266	−*73%*
PR – II	1535	570	−*18%*	8.4	0.4	*0.2*	707	484	−*61%*
Gr – II	1388	746	−*25%*	7.4	0.3	−*0.8*	491	208	−*73%*
Gr – III	921	467	−*52%*	7.8	0.4	*0.3*	221	128	−*73%*

Table 3 shows the average concentrations and rates of removal of NH_3_-N and production for NO_3_-N for each filter during Periods II & III. The full nitrogen speciation concentrations can be seen in Table A.2 in the SI. The difference between NH_3_-N removal and NO_3_-N production is the amount of NH_3_-N that was either lost from the system through adsorption or was not fully nitrified and exited as nitrite. The greatest difference between these values was in the GAC and Zeo filters. The GAC NO_2_-N concentration was 38 mg N L^−1^ during Period II, and the fresh GAC and washed GAC in Period III both released less than 8 mg N L^−1^. The Zeo NO_2_-N concentration was 171 mg N L^−1^. The summation of N species from these filters does not account for all NH_3_-N removed from the influent; therefore, some total N was removed from solution. Zeolite had the most removal of total N, but also released the greatest concentration of NO_2_-N, indicating incomplete nitrification.

**Table 3:** Average influent and effluent NH_3_-N, and NO_3_-N in trickling filters over Period II and III. The NH_3_ Δ is the removal from influent to effluent (reduction in concentration yields a negative Δ). The %_NH3_ value for nitrate is the percent of influent NH_3_-N recovered as NO_3_-N produced.

	NH_3_-N (mg/L)				NO_3_-N (mg/L)			
	Avg.	St. Dev.	*Δ (%)*	Removal rate (kg N/m^3^-d)	Avg.	St. Dev.	*(%*_*NH3*_*)*	Production rate (kg N/m^3^-d)
Influent – II	728	311	–	–	56	50	–	–
Influent – III	502	85	–	–	51	54	–	–
BC – II	138	47	−*81%*	0.100	569	171	*70%*	0.087
BC – III	56	70	−*89%*	0.075	381	184	*66%*	0.056
BC new - III	107	114	−*79%*	0.067	359	184	*61%*	0.052
GAC – II	125	81	−*83%*	0.102	530	113	*65%*	0.080
GAC – III	50	57	−*90%*	0.076	223	137	*50%*	0.029
GAC new – III	123	113	−*76%*	0.064	304	160	*34%*	0.043
Zeo – II	125	47	−*83%*	0.102	366	215	*43%*	0.052
PR – II	473	360	−*35%*	0.043	136	105	*11%*	0.014
Gr – II	172	85	−*76%*	0.094	472	176	*57%*	0.070
Gr – III	258	132	−*49%*	0.041	110	55	*12%*	0.010

**Fig. 1:**
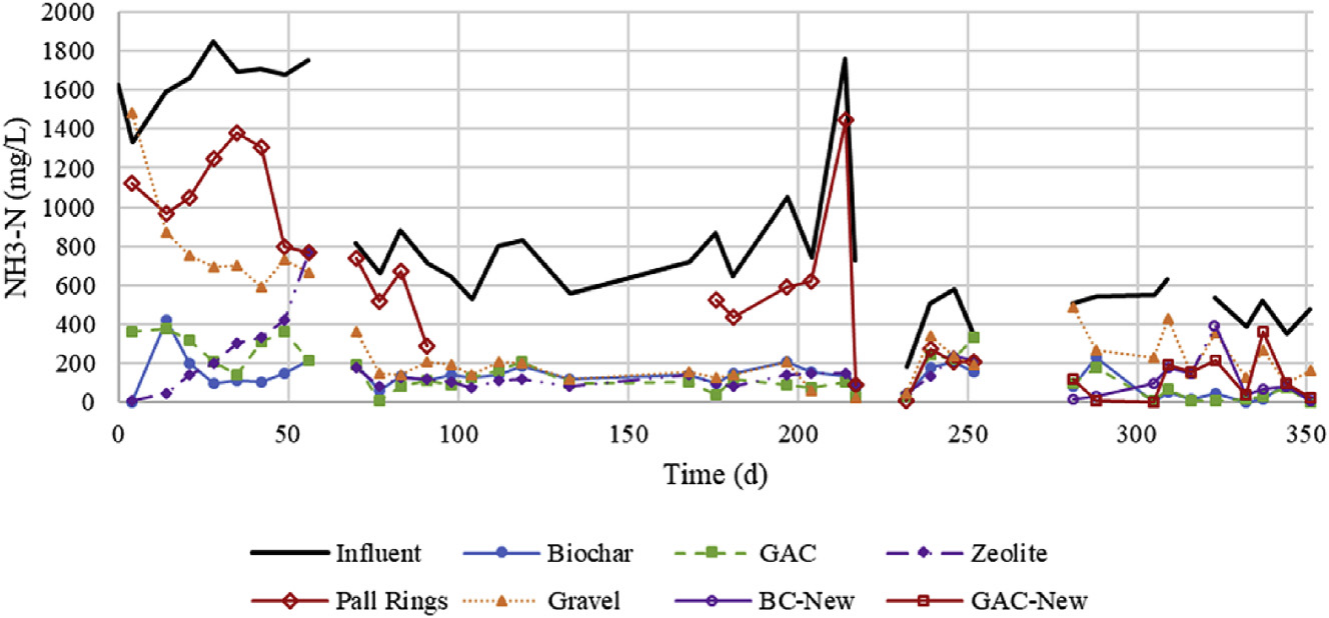
Total ammonia-nitrogen concentrations of influent and effluent of each trickling filter. Days 0–56 were Period I (start-up), days 63–252 were Period II, and days 281–351 were Period III (washed and new media).

**Fig. 2:**
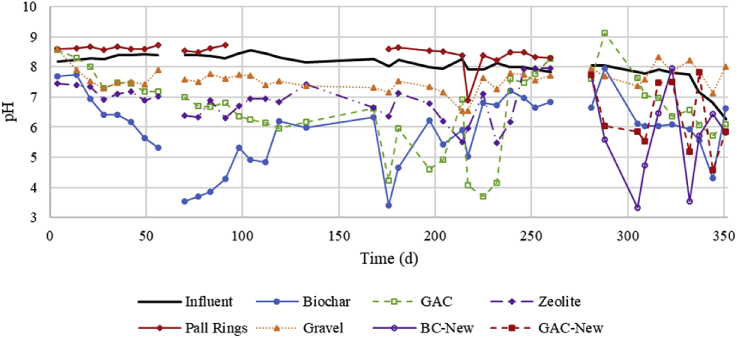
pH of influent and effluent of each trickling filter for the duration of the study.

Fig. 1, Fig. 2 show the NH_3_-N concentration and pH in the influent and effluent of each filter while turbidity data are shown in Figure A.1 in SI. The decrease in NH_3_-N and pH in the majority of filters during Period I (first 60 days) is indicative of the build-up of the nitrifying community.

The filters had stable performance from the start of Period II (day 63) until day 239. During this time, average turbidity removal for BC, GAC, Zeo, PR, and Gr was 90, 91, 77, 74, and 74%, respectively, and NH_3_-N removal was 83, 87, 85, 30, and 80%, respectively. Treatment performance began to decline after this time. Filters were then decommissioned to be washed with tap water. Zeolite and Pall rings were replaced with fresh GAC and biochar, which were deemed more promising media. Washing the media did not result in a long re-start period for BC and GAC filters. The trickling filter packed with gravel did exhibit a reduced performance after washing when comparing the end of Period II and start of Period III. This is likely due to the biofilm being washed off resulting in reduced removal rates. Performance for all washed media at the start of Period III was better than Period I, and the performance of washed BC and GAC was better than new BC and GAC. The higher removal rates were likely due to residual microbes and biofilm in media pores that were not washed off. Based on this finding, washing filter media every 6 months may yield the best results.

### 3.2 Denitrifying filters

Tracer tests showed that the retention time in S, B, B+S, E, and E+S filters was 36.7, 53.8, 32.9, 15.8, and 25.5 h, respectively. The measured retention time to theoretical retention time ratios for these filters were 0.51, 0.75, 0.46, 0.22, and 0.34, respectively. Table 4 reports average values, standard deviation, and percent change of denitrifying filter influent, mid-depth, and final effluent concentrations over the study period for COD, pH, and turbidity while Table 5 shows the total nitrogen, nitrate, and nitrate removal for the different filters. The performance was the highest in bamboo filters where TN removal rates were 2.6 and 1.4 times greater than sand only and eucalyptus filters, respectively.

**Table 4:** Average influent and effluent COD, pH, and turbidity for the submerged filters.

	COD (mg/L)			pH (−)			Turbidity (NTU)		
	*Avg.*	*St. Dev.*	*Δ (%)*	*Avg.*	*St. Dev.*	*Δ (%)*	*Avg.*	*St. Dev.*	*Δ (%)*
Influent	949	541		7.4	0.6		364	284	
Sand-mid	683	480	−*28%*	7.8	0.4	*6%*	57	48	−*84%*
Sand-final	479	290	−*50%*	7.5	0.4	*3%*	44	37	−*88%*
Bamboo-mid	587	286	−*38%*	7.7	0.3	*4%*	45	40	−*88%*
Bamboo-final	477	257	−*50%*	7.4	0.6	*0%*	40	35	−*89%*
B+S-mid	641	329	−*32%*	7.7	0.4	*4%*	59	47	−*84%*
B+S-final	505	296	−*47%*	7.6	0.3	*3%*	59	51	−*84%*
Eucalyptus-mid	533	279	−*44%*	7.4	0.4	*1%*	54	43	−*85%*
Eucalyptus-final	404	287	−*57%*	7.6	0.7	*3%*	40	37	−*89%*
E+S-mid	542	404	−*43%*	7.6	0.4	*4%*	55	48	−*85%*
E+S-final	434	235	−*54%*	7.5	0.3	*3%*	44	41	−*88%*

**Table 5:** Average influent and effluent TN and NH_3_-N for the submerged filters with normalized rates of removal.

	TN (mg/L)				NO_3_-N (mg/L)			
	*Avg.*	*St. Dev.*	*Δ (%)*	Removal rate (kg N/m^3^-d)	*Avg.*	*St. Dev.*	*Δ (%)*	Removal rate (kg N/m^3^-d)
Influent	405	134	–	–	299	79	–	–
Sand-mid	384	111	−*5%*	0.015	250	74	−*16%*	0.033
Sand-final	334	133	−*18%*	0.024	223	70	−*30%*	0.030
Bamboo-mid	278	75	−*32%*	0.086	167	64	−*44%*	0.089
Bamboo-final	223	86	−*45%*	0.062	103	61	−*66%*	0.066
B+S-mid	324	104	−*20%*	0.055	217	61	−*27%*	0.055
B+S-final	278	140	−*31%*	0.043	141	63	−*53%*	0.054
Eucalyptus-mid	318	96	−*22%*	0.059	237	72	−*21%*	0.042
Eucalyptus-final	277	94	−*32%*	0.043	195	64	−*35%*	0.036
E+S-mid	334	84	−*18%*	0.048	219	67	−*27%*	0.054
E+S-final	304	105	−*25%*	0.034	183	72	−*39%*	0.039

**Fig. 3:**
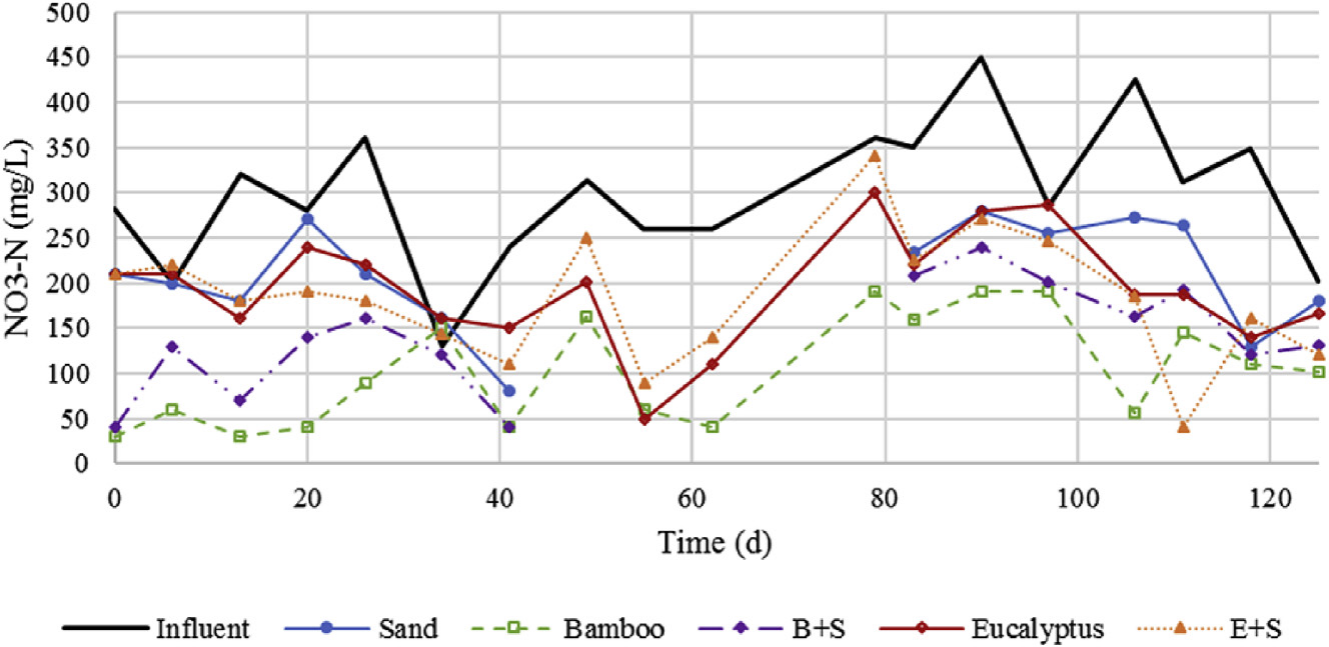
Influent and filter effluent NO_3_-N concentration for each media type over the denitrifying filter study period.

Fig. 3 displays the influent and filter effluent concentrations of NO_3_-N for each media type during the study period. There are no data points for sand and B+S filters from day 49–70 as there was regular clogging at the surface of these filters during that time. The top 1 cm of these filters was scraped and removed on day 70, and thereafter, the top surface of these filters was stirred weekly to induce free flow to remedy this issue. Effluent NO_3_-N concentrations from the bamboo-only filter consistently remained the lowest while effluent turbidity was similar in all filters. Turbidity removal was consistently high throughout the study, and effluent turbidity decreased moderately over time as the filters aged (see Figure A.3 in SI). The average influent NO_2_-N concentration was 14.8 mg L^−1^ and reduced to 0.5–3.7 mg L^−1^ (i.e., at least 75% removal) in all filters. The bamboo filters had the lowest concentrations in their effluent (1.1 mg L^−1^ in B and 0.5 mg L^−1^ in B+S) (Table A.2 in SI).

### 3.3 Wood chip COD release

**Fig. 4:**
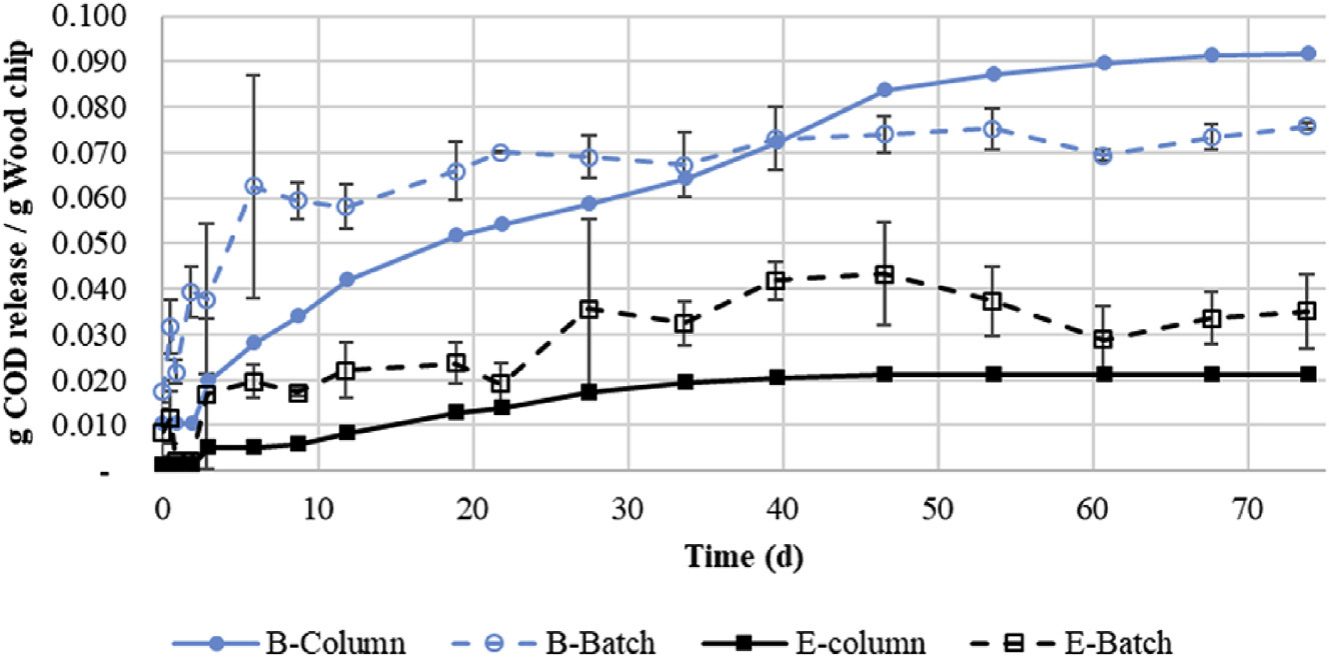
Total cumulative COD released per gram (wet weight) of wood chips (B=Bamboo and E = Eucalyptus) in continuously-fed column and in batch experiments. Error bars are the standard of deviation between the samples tested in triplicate.

The amount of COD released by bamboo and eucalyptus during the batch experiment is shown in Fig. 4. One of the flasks for bamboo exploded on day 18 due to high gas pressure. COD release from bamboo was analyzed in duplicate after this time. Greater scattering of the results was noticed for the tests with eucalyptus after day 21. Slow denitrification rates were seen until this time, but two of the three bottles exhibited rapidly increased COD release after this time while the third remained at a slow rate. Some dips in average cumulative COD release can be seen in Fig. 4; they are the result of experimental uncertainties. In general, the error bars overlap for points before and after dips. Because of these dips, the maximum value instead of final value of the COD released was best used for analysis. The maximum COD release for bamboo was $\text{0}\text{.}076\;{\text{g}}_{\text{COD}}\;{\text{g}}_{{\text{bamboo}}^{\mathrm{-1}}}$, while the maximum COD released for eucalyptus was $\text{0}\text{.}043\;{\text{g}}_{\text{COD}}\;{\text{g}}_{\text{TS,}\;{\text{eucalyptus}}^{\mathrm{-1}}}$. If COD release was normalized by gram TS (bamboo 94% TS, eucalyptus 36%), the maximum releases were $\text{0}\text{.}080\;{\text{g}}_{\text{COD}}\;{\text{g}}_{\text{TS,}\;{\text{bamboo}}^{\mathrm{-1}}}$ and $\text{0}\text{.}119\;{\text{g}}_{\text{COD}}\;{\text{g}}_{\text{TS,}\;{\text{eucalyptus}}^{\mathrm{-1}}}$. In terms of future design, however, COD release per wet mass may be the more practical parameter than dry mass.

The total COD release and rate of release from bamboo were both significantly greater than for eucalyptus. This is consistent with the observation that the rate of nitrate removal was much faster in bamboo flasks than in eucalyptus flasks. No nitrate remained in bamboo flasks after 8 days, but 73 days were required to remove nitrate in eucalyptus flasks (one of the three flasks still had 87 mg NO_3_-N L^−1^ on this day) (see additional results in Figure A.3).

Fig. 4 also displays the COD released during the continuously-fed column experiment. Similar to the batch experiment, the bamboo wood chips released more COD per gram of wood. The total amount released was $\text{0}\text{.}092\;{\text{g}}_{\text{COD}}\;{\text{g}}_{{\text{bamboo}}^{\mathrm{-1}}}$ and $\text{0}\text{.}020\;{\text{g}}_{\text{COD}}\;{\text{g}}_{{\text{eucalyptus}}^{\mathrm{-1}}}$. In terms of release per gram of TS, the values were $\text{0}\text{.}098\;{\text{g}}_{\text{COD}}\;{\text{g}}_{\text{TS,}\;{\text{bamboo}}^{\mathrm{-1}}}$ and $\text{0}\text{.}083\;{\text{g}}_{\text{COD}}\;{\text{g}}_{\text{TS,}\;{\text{eucalyptus}}^{\mathrm{-1}}}$. The amounts of COD released during the continuously-fed column were comparable to those of the batch experiment.

## 4 Discussion

### 4.1 Trickling filters

#### 4.1.1 Start up

An indicator of nitrification is change in pH as 2 mol of H^+^ are released per mole of NH_4_^+^ oxidized to nitrite, and the decline of pH over time can be interpreted as the time for the nitrifying community to build up. As seen in Fig. 2, the pH dropped the most for the BC filter, followed by GAC and Zeo filters. The pH for most filters leveled off after 28 days, suggesting this was the time required for the nitrifying community to stabilize under these loadings. This start-up time was similar to the 20–30 days required for nitrifying trickling filters as previously seen (Cai et al., 2015; Li et al., 2016). As seen with other indicators, such as NO_3_-N increase, the BC filter exhibited the greatest reduction of NH_3_ by nitrification. Another observation during the start-up period is the low concentrations of NH_3_ in the effluent from the beginning for the BC, GAC, and Zeo filters, even before the nitrifying community developed as indicated by pH (Fig. 1). This decrease of NH_3_ concentration can be interpreted by adsorption to these media, which was expected based on their properties. In contrast, NH_3_ effluent concentrations from the gravel (which has no N sorption capacity) filter slowly decreased, consistent with the need for the nitrifying community to build up for NH_3_ removal.

#### 4.1.2 Normal operation

The BC and GAC filters showed the best performance in reducing NH_3_. The performance of these two filters were similar for the duration of the experiment (see Fig. 1) with NH_3_ removal in the GAC filter being generally slightly better, possibly because of slightly better adsorption capacity. The filter packed with gravel showed an opposite trend, improving in performance with time; the smooth surface of the gravel certainly caused the biofilm to form more slowly than onto the more porous supports. Additionally, gravel has no absorption or adsorption capacity like BC, GAC, and zeolite. The filter packed with Pall rings performed the worst, most likely due to the short liquid retention time and limited biofilm growth on the plastic packing.

Biochar and GAC filters provided similar ammonium removal performance over both Period II and III. The primary difference between the two was that the filter packed with GAC removed more TN than the filter packed with biochar. This removal is hypothesized to be due to adsorption in the GAC which is made up of micropores (more so than biochar) that are effective in adsorbing dissolved contaminants, such as ammonium (Simpson, 2008). These micropores, however, become blocked by suspended solids and biofilm growth, limiting adsorptive capacity when treating high-strength waste (Huggins et al., 2016). Reductions in NH_3_-N concentrations were similar in both periods; however, increases in NO_3_-N concentrations were greater in the BC filter than in the GAC one. Decreased absorptive capacity was displayed as the nitrate production was greater in GAC filters used for a longer duration (Table 3,GAC-II). Further, the pH decrease shown in Fig. 2 for GAC-II indicates increased nitrification over time while ammonium removal remained relatively constant (Fig. 1). Additionally, a significant difference was displayed in nitrate production in Phase III between the washed GAC and fresh GAC. This can be explained by the fact that washed GAC would not reactivate micropores and begin with a lower adsorptive capacity than fresh GAC. Therefore, while nitrification and adsorption both reduce NH_3_-N concentrations in each filter, adsorption likely played a greater role in the GAC column.

In comparison to filters with BC and GAC, NH_3_ concentrations in the effluent of the Zeo filter rapidly increased at the end of the startup phase (days 35–56), followed by a decrease of effluent NH_3_ concentrations during Period II to reach levels similar to that of filters packed with BC & GAC. This increase and then decrease in effluent ammonium concentration is attributed to filling adsorption sites during startup and a longer time to build up the nitrification community. Later, from days 133–252, the filter packed with Zeo had similar effluent NH_3_ but much lower NO_3_ concentrations than the BC and GAC filters. This signifies a shift in NH_3_ removal mechanism from ion exchange (during Period I) to nitrification and back to ion exchange (during Period II). This dynamic is likely related to the ability for zeolite to be regenerated as nitrifying bacteria consume NH_4_^+^, as was found by Aiyuk et al. (2004).

Overall, most of the trickling filters showed high NH_3_-N removal rates. With the exception of the Pall ring filter, all filters showed the ability to achieve nearly 0.10 kg N m^−3^ d^−1^. The rates achieved in this study are significantly higher than found in a previous study testing both biochar and GAC (approximately 0.03 kg NH_3_-N m^−3^ d^−1^ each) (Berger, 2012). The high removal rate values could be due to the high inlet concentrations (800 mg N L^−1^ vs 95 mg N L^−1^ for the Berger study), which also show that these filters can treat high strength waste.

Nitrification has a significant oxygen demand (4.57 g O_2_/g NH_4_^+^-N (Rittmann and McCarty, 2001)). Our trickling filters were operated with passive aeration using a natural draft which was sufficient as seen by high nitrification rates. Measured DO (Figure A.2) confirmed adequate oxygenation as the DO concentration increased from 0.8 mg_DO_ L^−1^ in the substrate influent to 5 mg_DO_ L^−1^ in BC and GAC filter effluent. Proper aeration would have to be re-assessed in full-scale application, however, as air draft is size and geometry dependent. The narrow columns (1 m length x 10 cm dia.) in this experiment are more conducive to natural draft than a full-scale system which a much larger diameter.

### 4.2 Submerged filters

Denitrification performance was much greater over the first two months than for the next two months. From day 1–62, the average NO_3_-N concentration in the influent of the anaerobic filters was 265 mg-N L^−1^, and outlet concentrations for S, B, B+S, E, and E+S were 187, 70, 100, 171, and 171 mg NO_3_-N L^−1^ with corresponding removals of 29, 73, 64, 35, and 35%, respectively. The NO_3_-N loading did not significantly change from the first half to the second half of the study, however, the inlet COD concentration decreased from first half to second half (1150 to 730 mg_COD_ L^−1^) which may have affected the denitrification performance. Another possible reason for decreased performance could be a partial exhaustion of the most available carbon released by the wood chips; however, there was no increasing trend of effluent nitrate concentrations towards the end of the study, showing that the wood chips were still providing reducing equivalents for denitrification at that time.

The COD supplemented by wood chips to the submerged filters had the potential to increase COD concentration in filter effluent; however, effluent COD did not significantly vary between filters. The average outlet concentrations were nearly the same, 479 and 477 mg_COD_ L^−1^ for S and B, respectively. In terms of BOD (3 sampling events), filters with wood chips had lower effluent concentrations than the ones packed with sand (123, 54, 62, 53, and 45 mg_BOD_ L^−1^ for S, B, B+S, E, E+S; influent was 147 mg_BOD_ L^−1^). BOD samples were taken on days 41, 106, and 132. S, E, and E+S filters had increasing BOD concentrations while B and B+S filter did not show either an increasing or decreasing trend in concentration. The similar COD effluent concentrations were likely due to high nitrate removal performance in filters with wood chips. The COD released by the wood chips was consumed by denitrifiers, and the COD remaining in the effluent was mostly not bioavailable. Based on these results, the likely limiting factor in filter performance was having sufficient electron donor for denitrification, which is consistent with general denitrification operation in that the electron donor is generally the rate-limiting substrate (Rittmann and McCarty, 2001). Additional evidence of this hypothesis is that sand filter had much lower nitrate removal while having the same COD loading as other filters.

The highest nitrate removal rates were seen in the bamboo wood chips column, 0.089 and 0.066 kg N m^−3^ d^−1^ at mid-depth and outlet, respectively. Since nitrate removal in the sand filter is supported by the consumption of COD in the inlet, the nitrate removal linked to COD released by the bamboo only can be found by difference and was 0.056 and 0.036 kg N m^−3^ d^−1^, respectively. This rate is higher than a previous study using eucalyptus wood chips that found a rate of 0.014 kg N m^−3^ d^−1^ (Lynn et al., 2015a). Eucalyptus wood chips in our study, however, did not perform as well. The nitrate removal rates for the eucalyptus-only filter calculated from mid-depth and final outlet concentrations were 0.042 and 0.036 kg N m^−3^ d^−1^, respectively. While this is higher than the previous study by Lynn et al., 2015a, 2015b the removal rate in the sand-only filter was similar (0.030 kg N m^−3^ d^−1^ across the whole filter), showing that eucalyptus did not significantly contribute to denitrification in terms of supplementing electron donors. Removal rates in all filters were greater in the first half of the filter than in the second half. The higher rate in the first half of the filter supports the finding of Aslan (2008) that the majority of nitrate removal occurs in the top portion of the filter bed in a downflow reactor. If the bamboo- and eucalyptus-only reactors were to be resized based on their overall removal rates to completely denitrify an influent loading of 300 mg NO_3_-N L^−1^ at 1.35 L d^−1^, the resulting volumes would be 6.1 L for bamboo and 11.3 L for eucalyptus.

At a flow of 1.35 L d^−1^, the sand filter removed 635 mg COD d^−1^ and 122 mg NO_3_-N d^−1^. By using sand as the control for the amount of COD from the influent consumed for denitrification, the COD provided by the bamboo and eucalyptus filters for denitrification can be predicted. B, B+S, E, and E+S filters removed 265, 215, 143, and 156 mg NO_3_-N d^−1^, respectively. The COD:NO_3_^−^ ratio in the sand filter was 5.2:1. Using that same ratio for NO_3_-N removal in other filters yields 1,380, 1,120, 740, and 810 mg COD d^−1^ consumed for denitrification in the B, B+S, E, and E+S filters, respectively of which 749, 487, 110, and 179 mg COD d^−1^ came from the wood chips. The B filter had 978 g bamboo, resulting in a release rate of 0.765 mg_COD_ g_bamboo_^−1^ d^−1^, and the E filter had 1070 g eucalyptus, resulting in 0.103 mg_COD_ g_eucalyptus_^−1^ d^−1^.

The calculated rate of COD release from bamboo wood chips was less in the treatment study (0.765 mg_COD_ g_bamboo_^−1^ d^−1^) than found in the column (1.26 mg_COD_ g_bamboo_^−1^ d^−1^) and batch studies (1.04 mg_COD_ g_bamboo_^−1^ d^−1^). However, COD release plateaued in column and batch studies by day 73, while the COD release rate in the treatment study had still not plateaued (see Fig. 4) by the end of the treatment study period (123 d). The total release in the treatment, column, and batch studies according to study duration was 0.094 g_COD_ g_bamboo_^−1^, 0.092 g_COD_ g_bamboo_^−1^, and 0.076 g_COD_ g_bamboo_^−1^, respectively. These findings show that the bamboo wood chips in the column study for COD release produced a greater total COD release as well as release rate than in the batch study while the greatest total COD release for bamboo wood chips was in the actual treatment study. The reason for these findings may be that the continuous feed of substrate (nitrate) in the column study enhanced microbial activity, either directly extracting more COD out of the wood chips or promoting hydrolysis of the wood chips.

Total eucalyptus COD release as well as release rate was lower in the treatment study (0.013 g_COD_ g_eucalyptus_^−1^ and 0.103 mg_COD_ g_eucalyptus_^−1^ d^−1^) than in the column study (0.030 g_COD_ g_eucalyptus_^−1^ and 0.411 mg_COD_ g_eucalyptus_^−1^ d^−1^) and batch study (0.043 g_COD_ g_eucalyptus_^−1^ and 0.589 mg_COD_ g_eucalyptus_^−1^ d^−1^). This opposite relationship for eucalyptus is unclear, but the greater release in the batch experiment than in the column experiment may be due to the shorter retention time compared to column with bamboo, and due to slow hydrolysis.

Turbidity removal performance was nearly identical across filters (84–88%). Bamboo and eucalyptus wood chip filters showed less turbidity removal which was attributed to higher void space compared to sand, which is why two filters were tested using a combination of sand and wood chips. However, wood chips plus sand filters did not show any significant improvement in turbidity removal. The use of sand resulted in decreased performance over time as clogging was experienced in sand and B+S filters by day 49 and day 62 for E+S. From this time on, the top layer of the filter was stirred weekly to disturb buildup and induce free flow. With this scenario, bamboo wood chips alone were the overall best performing filters by providing the greatest nitrate removal without losing turbidity removal performance while having greater longevity. Biochar could also be used as an alternative medium to improve filtration and increase nitrate removal as has been suggested by Bock et al. (2015, 2016). These studies found increased nitrate removal by amending wood chips with 10% biochar by volume when influent nitrate concentrations are high.

### 4.3 Combined filter performance

A desired outcome of this study was to inform a possible future implementation for effluent treatment of onsite sanitation systems such as those described by Forbis-Stokes et al. (2016). Based on the current study, the chosen media would be biochar for the trickling filter and bamboo for the submerged denitrifying filter because of their high performance as well as widely available and sustainable procurement. By keeping the design and loading conditions the same with average influent concentrations of 600 mg NH_3_-N L^−1^, 50 mg NO_3_-N L^−1^, and 980 NTU and 6 d EBRT, the biochar filter's expected effluent would be 97 mg NH_3_-N L^−1^, 475 mg NO_3_-N L^−1^, and 120 NTU. The bamboo filter (3 d EBRT) would then produce an effluent of 82 mg NH_3_-N L^−1^, 157 mg NO_3_-N L^−1^, and 13 NTU. This theoretical combined performance would thus result in 63% removal of total N and 99% removal of turbidity. The limiting step to greater total N reduction in this setup would be denitrification. If more N removal is required, increasing the volume of the bamboo denitrifying filter is likely to improve performance as the electron donor was demonstrated to limit denitrification. Based on the observed nitrate removal rates, the bamboo filter volume would need to be increased to 7.2 L (or a volumetric loading of 0.19 m^3^ m^−3^ d^−1^) to accommodate the nitrate loading. In this scenario, total nitrogen removal efficiency of 80–90% will likely be achieved. Another approach to improve denitrification is to pre-treat wood chips to promote hydrolysis of the cellulosic structure. Particle size reduction, thermal treatment, acidic treatment, and alkali treatment are all methods of improving the biodegradability of lignocellulosic materials (Montgomery and Bochmann, 2014) that may be beneficial in this application.

The filter systems that were investigated required minimal maintenance. Trickling filters were in operation for about 6 months before clogging became an issue. When media was removed and washed with tap water, full treatment performance was recovered almost instantly and required less start-up time than in the initial start-up period. Submerged filters with sand clogged within 2 months of operation while filters without sand did not experience clogging in the 4-month study period. Additionally, no increasing nitrate effluent concentration trend was seen at the end of the study period, showing that the wood chips were still supplementing electrons for denitrification at that time. Based on these findings, a 6-month maintenance interval for trickling filters, and a 4- or up to 6-month maintenance interval for bamboo denitrifying filters are recommended.

Another consideration for filter performance is phosphorus removal which is not included in the previous discussion as the focus of this work was on nitrogen removal. The phosphorus removal efficiencies (measured as PO_4_^3−^) in the trickling filters with BC, GAC, and Zeo were similar and ranged from 31 to 42%. Removal in the denitrifying filters ranged from 27% for the eucalyptus filter to 41% in the all-sand filter. The primary mechanism of phosphorus removal in this experiment is hypothesized to be precipitation and filtration and not biological removal which is consistent with the observation that the sand denitrifying filters performed better than those without sand. Additionally, the operational condition for these filters was unfavorable for biological phosphorus removal. For phosphorus treatment, two methods of interest are (1) incorporating media into the denitrifying filters such as iron (Erickson et al., 2012) or biochar amended wood chips (Bock et al., 2015; Pluer et al., 2016) for combined physical and chemical phosphate removal, or (2) phosphorus precipitation downstream of the denitrifying filter by adding magnesium to produce struvite (Etter et al., 2011) or flowing through steel slag (Christianson et al., 2017). Further research is required to quantify and optimize phosphorus removal.

## 5 Conclusion

This study evaluated the use of various packing materials as nitrification and denitrification media for the treatment of high-strength anaerobic digester effluent. Maximum ammonia-nitrogen removal (either nitrification or total N removal) in filters with biochar and GAC reached 81–90% (at an influent concentration of 600 mg NH_3_-N L^−1^). These same filters achieved 83–94% removal of turbidity while treating a waste stream with approximately 1000 NTU. Investigations with submerged attached growth filters evaluated the use of sand, bamboo, bamboo with sand, eucalyptus, and eucalyptus with sand as denitrification systems for treating the effluent of the nitrification trickling filters. The maximum nitrate removal was 66% of NO_3_-N and 89% removal of turbidity in the filter packed with bamboo. The trickling and submerged filters showed high removal rates while treating high concentrations of ammonia and nitrogen and, thus, offer promising post-treatment options for digester effluent for simultaneous organic and nitrogen removal. These systems required minimal attention as estimated time between required maintenance was 4–6 months. Both filter stages provided dramatic improvement in effluent clarity as well as odor, as there was little to no detectable offensive odor in the effluent of either filter. The improved aesthetics of the treated effluent may allow for water reuse (e.g., for toilet flushing). The trickling filter produces an effluent with high total nitrogen concentrations which could be reused as fertilizer if only this first stage of treatment was used. If local fertilizer reuse is not desired, the second stage denitrification filter can be used. If adequately sterilized, the second stage denitrification filter effluent could potentially be reused as flushing water or wash water, or be safely discharged in the environment, depending on local regulations.

## Author disclosure statement

The authors declare that they have no conflicts of interest or competing financial interests.

## Appendix A Supplementary data

mmc1 mmc1
