# Supplementary material for: Nitrifying trickling filters and denitrifying bioreactors for nitrogen management of high-strength anaerobic digestion effluent
Source: Chemosphere. 2018 Aug;204:119–29. doi: 10.1016/j.chemosphere.2018.03.137 (PMC5953278; doi:10.1016/j.chemosphere.2018.03.137)
Supplement: mmc1 [file mmc1.docx]

**Supporting Information to**

**Nitrifying Trickling Filters and Denitrifying Bioreactors for Nitrogen Management of High-Strength Anaerobic Digestion Effluent**

Aaron A. Forbis-Stokes^a,b^, Lucas Rocha-Melogno^a^, Marc A. Deshusses^a,b*^

^a^ Department of Civil & Environmental Engineering, Duke University, Durham, NC

^b^ Duke Global Health Institute, Duke University, Durham, NC

**Corresponding author*: Department of Civil & Environmental Engineering, Duke University, 127C Hudson Hall, Box 90287, Durham, NC 27708. *Phone:* (919) 660-5480; *Fax*: (919) 660-5219.*; emails:* marc.deshusses@duke.edu, aaron.forbis-stokes@duke.edu

Additional media analysis results.

**Table A.1.** Particle size distribution for the filter media. Particle size distribution is provided
according to grain size in U.S. Standard mesh number and in mm. Pall rings were not included
in the table because of their uniform size of 16 mm.

| Grain size (#) | Grain size (mm) | Percent distribution (%) | | | | | | |
| --- | --- | --- | --- | --- | --- | --- | --- | --- |
|  |  | Biochar | GAC | Zeolite | Gravel | Sand | Bamboo | Eucalyptus |
| >1/4" | >6.35 | 10.6% | 0.0% | 1.1% | 82.2% |  | 0.0% | 0.0% |
| #4-1/4" | 4.75-6.35 | 84.6% | 2.0% | 35.8% | 16.8% |  | 81.3% | 49.0% |
| #4-6 | 3.35-4.75 | 3.2% | 45.5% | 42.1% | 1.0% |  |  |  |
| #6-8 | 2.35-3.35 | 1.1% | 50.5% | 20.0% | 0.0% |  | 7.4% | 36.5% |
| #8-16 | 1.18-2.36 | 0.4% | 2.0% | 1.1% | 0.0% |  | 6.4% | 7.4% |
| #16-20 | 0.710-1.18 | 0.2% | 0.0% | 0.0% | 0.0% | 4.1% | 1.5% | 2.2% |
| #20-40 | 0.425-0.710 |  |  |  |  | 55.1% | 2.3% | 3.6% |
| #40-60 | 0.250-0.425 |  |  |  |  | 30.6% | 0.6% | 0.9% |
| #60-80 | 0.180-0.250 |  |  |  |  | 5.1% | 0.2% | 0.1% |
| #80-140 | 0.105-0.180 |  |  |  |  | 3.1% | 0.4% | 0.3% |
| <#140 | <0.105 |  |  |  |  | 2.0% | 0.0% | 0.0% |


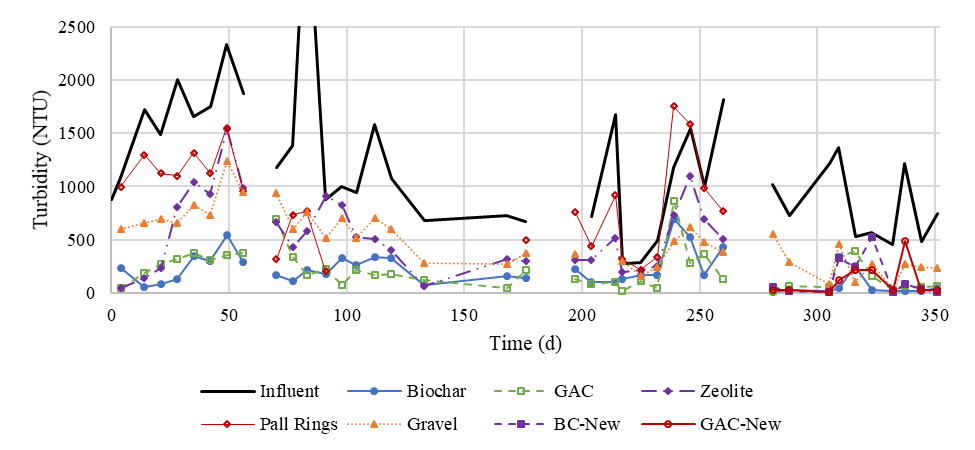


**Figure A.1.** Turbidity (NTU) of filter influent and effluent from each filter for the duration of the study.


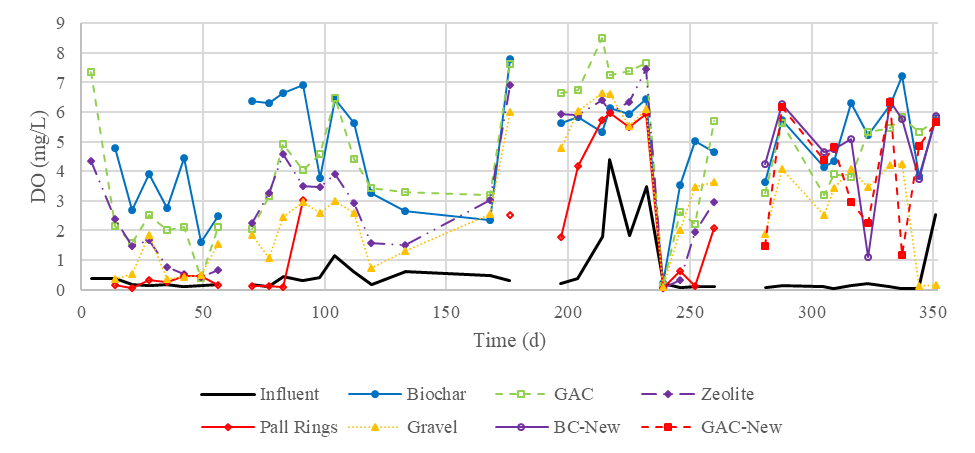


**Figure A.2.** Influent and effluent dissolved oxygen (DO) concentrations for
each filter in Period II and III.


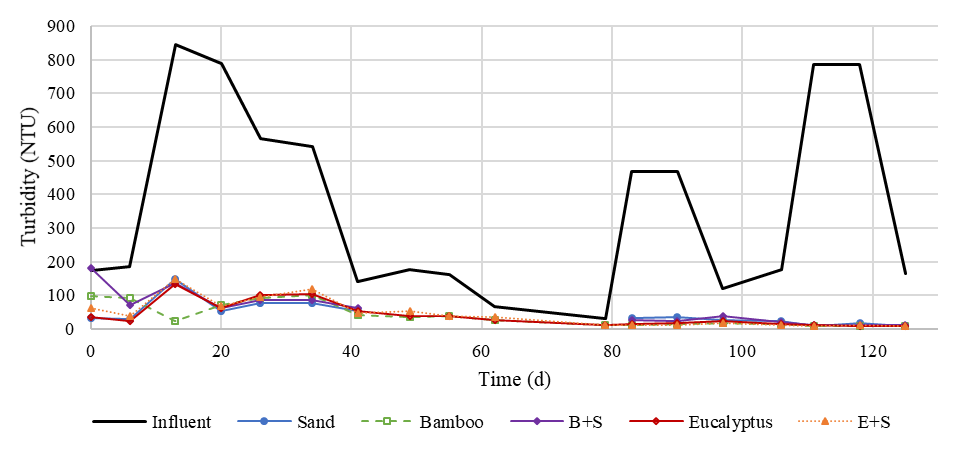


**Figure A.3.** Influent and filter effluent turbidity for each media type over
the denitrifying filter study period.


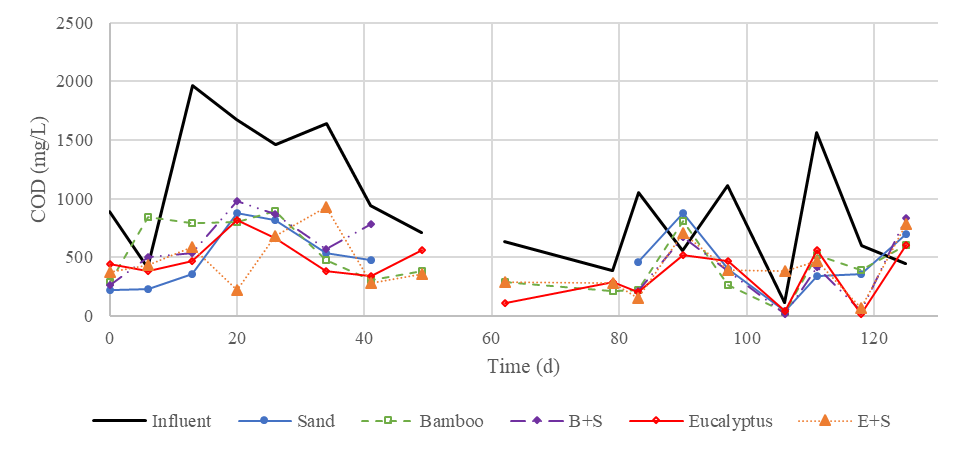


**Figure A.4.** Influent and effluent COD concentrations for each denitrifying filter.

**Table A.2.** Average influent and effluent nitrogen speciation in nitrification and denitrification
filters for the full study duration.

|  | TN (mg/L) | | | NH_3_-N (mg/L) | | NO_3_-N (mg/L) | | | NO_2_-N (mg/L) | | | |
| --- | --- | --- | --- | --- | --- | --- | --- | --- | --- | --- | --- | --- |
|  | Avg. | | St. Dev. | Avg. | St. Dev. | Avg. | St. Dev. | | Avg. | | St. Dev. | |
|  | | Nitrification Filters | | | | | | | | | |  |
| Influent – II | 804 | | 298 | 728 | 311 | 56 | 50 | 3 | | 3 | |  |
| Influent – III | 667 | | 163 | 502 | 85 | 51 | 54 | 3.0 | | 3.6 | |  |
| BC – II | 663 | | 169 | 138 | 47 | 569 | 171 | 2 | | 2 | |  |
| BC – III | 431 | | 153 | 56 | 70 | 381 | 184 | 3.0 | | 6.6 | |  |
| BC new - III | 470 | | 192 | 107 | 114 | 359 | 184 | 0.7 | | 0.6 | |  |
| GAC – II | 604 | | 171 | 125 | 81 | 530 | 113 | 38 | | 99 | |  |
| GAC – III | 341 | | 108 | 50 | 57 | 223 | 137 | 7.9 | | 13.5 | |  |
| GAC new – III | 452 | | 150 | 123 | 113 | 304 | 160 | 0.7 | | 0.7 | |  |
| Zeo – II | 653 | | 192 | 125 | 47 | 366 | 215 | 171 | | 104 | |  |
| PR – II | 744 | | 295 | 473 | 360 | 136 | 105 | 39 | | 44 | |  |
| Gr – II | 683 | | 212 | 172 | 85 | 472 | 176 | 43 | | 53 | |  |
| Gr – III | 433 | | 171 | 258 | 132 | 110 | 55 | 20.6 | | 19.8 | |  |
|  | | Denitrification Filters | | | | | | | | | |  |
| Influent | 405 | | 134 | 128 | 89 | 299 | 79 | 14.8 | | 13.0 | |  |
| Sand-mid | 384 | | 111 | 120 | 120 | 250 | 74 | 4.6 | | 6.6 | |  |
| Sand-final | 334 | | 133 | 132 | 85 | 223 | 70 | 3.7 | | 6.2 | |  |
| Bamboo-mid | 278 | | 75 | 101 | 101 | 167 | 64 | 1.1 | | 1.1 | |  |
| Bamboo-final | 223 | | 86 | 109 | 83 | 103 | 61 | 1.2 | | 1.1 | |  |
| B+S-mid | 324 | | 104 | 120 | 120 | 217 | 61 | 0.5 | | 0.5 | |  |
| B+S-final | 278 | | 140 | 126 | 89 | 141 | 63 | 0.5 | | 0.5 | |  |
| Eucalyptus-mid | 318 | | 96 | 99 | 99 | 237 | 72 | 3.1 | | 4.0 | |  |
| Eucalyptus-final | 277 | | 94 | 82 | 68 | 195 | 64 | 3.1 | | 3.6 | |  |
| E+S-mid | 334 | | 84 | 120 | 120 | 219 | 67 | 2.9 | | 3.0 | |  |
| E+S-final | 304 | | 105 | 113 | 82 | 183 | 72 | 3.3 | | 4.5 | |  |


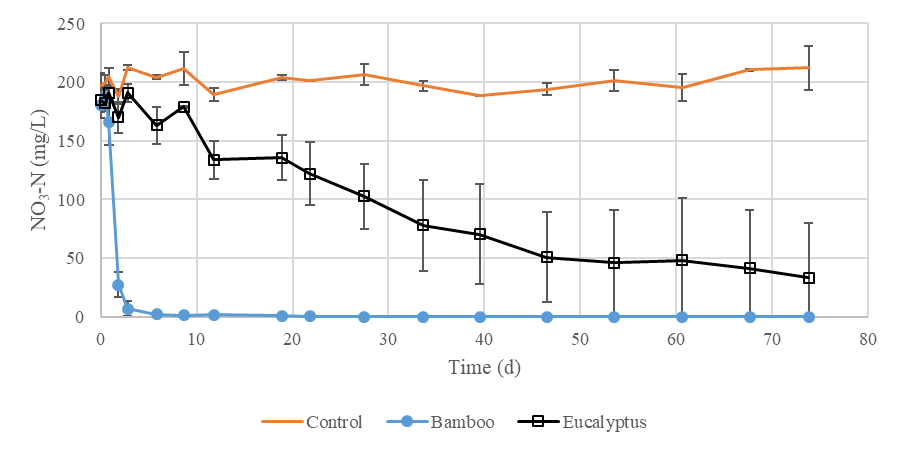


**Figure A.5.** NO_3_-N concentration of wood chip solution stored in flasks.
Error bars are standard deviations.


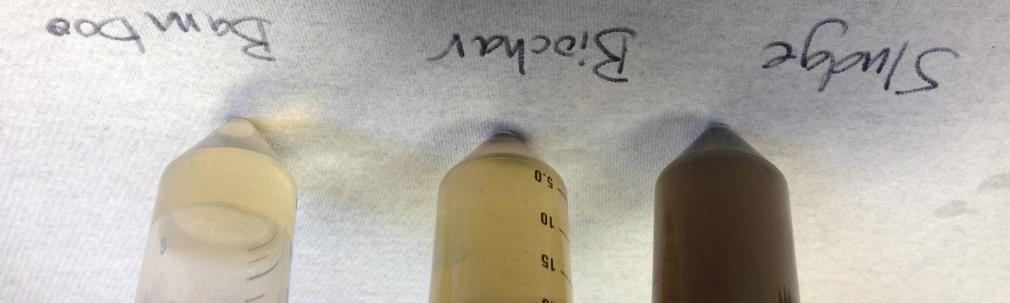


**Figure A.6.** Picture of samples taken from system influent (marked as “sludge”), and effluents from the biochar and bamboo filters.
